# Automatic chick cough detection system based on improved audio spectrogram convolutional transformer neural network

**DOI:** 10.3389/fvets.2026.1810310

**Published:** 2026-04-24

**Authors:** Bowen Cai, Bo Zhou, Xiangshuai Kong, Mengsi Zhai

**Affiliations:** 1 School of Environmental, Tsinghua University, Beijing, China; 2 Key Laboratory of Digital and Intelligent Technology Integration for Healthy Poultry Farming, Ministry of Agriculture and Rural Affairs, Nanjing, Jiangsu, China; 3 Shanghai Xiashu Intelligent Technology, Shanghai, China

**Keywords:** audio spectrogram transformer, cough detection, edge computing, local multi-head attention, poultry science, respiratory disease monitoring

## Abstract

Respiratory diseases are common on poultry farms during spring and autumn. Due to the high density of the farm environment, an epidemic can spread very quickly and cause large-scale biological infections. Therefore, developing software capable of monitoring or providing early warnings of respiratory disease in chickens is very important because it helps prevent the spread of diseases and enhances the health of the chickens. This study proposes an acoustic detection system for chicken coughing (ASCT-CC) designed for real-world poultry farming environments. This system is based on an improved audio spectrogram transformer (AST) architecture, using a hybrid convolutional-transformer backbone network that replaces the global attention mechanism with local multi-head attention. This architecture effectively improves the model’s ability to capture crucial local acoustic information and increases its robustness against noise at a lower computation cost. Besides, the study constructs a two-branch co-learning structure, adopts focal loss as an auxiliary strategy to reduce sample bias, and combines these with the connectionist temporal classification (CTC) decoder to accurately identify and temporally localize the cough event. To meet practical application requirements, the system is deployed with low latency on edge computing devices using TensorRT acceleration and INT8 quantization technology. Experiments demonstrated that our model achieves an mAP of 92.86% during training and reaches an identification rate of 92.11% on the independent test set, with an inference time of only around 200 ms. This system provides 24 h real-time monitoring and multi-level early warning capabilities, offering effective technical support for the early detection and intelligent control of respiratory diseases in poultry.

## 1 Introduction

Poultry farming, as a core component of the livestock industry, has experienced a continuous increase in stocking density and expansion in scale. However, while large-scale farming improves production efficiency, animal health management on farms is facing increasingly severe challenges. Respiratory diseases are among the most common and economically damaging diseases in poultry farming, with a rapid spread, a wide range of impacts, and high death losses. They are highly susceptible to rapid spread within a population, leading not only to decreased poultry growth performance, increased mortality, and higher treatment costs, resulting in significant industry losses (1), but also to the overuse of antibiotics, thereby triggering food safety and public health risks (2). In the current high-density breeding environment, early prevention and control of chicken respiratory disease are particularly important, as they directly affect the productivity of farms. Manual patrolling and inspection are the primary methods for monitoring diseases, making preliminary diagnoses through observation of the birds’ behavior and by listening for any unusual noises, such as coughing and sneezing.

The drawbacks and challenges of traditional methods are also apparent. On the one hand, these methods are highly subjective and heavily influenced by poultry farmers’ experience. Different farmers have varying sensitivities and judgments regarding coughing sounds, leading to missed detections and misjudgments. On the other hand, they do not provide real-time monitoring. Delayed inspections can lead to the spread of disease. Furthermore, they are costly in terms of workforce, and frequent manual inspections can pose biosecurity risks and stress to the poultry flock (3). Moreover, the complex environment inside poultry houses, often accompanied by various noise interferences such as fans, feeders, and poultry calls, further complicates manual identification and makes it difficult to meet the needs of large-scale farming scenarios (4). Currently, automated monitoring methods for poultry health status mainly include monitoring body temperature changes and fecal composition analysis (5). These methods can reflect the overall health level of individuals, but their correlation with respiratory diseases is mostly indirect, making it difficult to specifically identify respiratory infections. Conversely, coughing, as an important external symptom of respiratory diseases in poultry, can more directly reflect the pathological changes in the respiratory system. Related studies have shown that respiratory pathogen infection can trigger an inflammatory response, thereby altering the vibrational characteristics of the airflow channel and vocal organs, causing significant abnormalities in the spectral structure, energy distribution, and timing patterns of coughing sounds (6). Therefore, acoustic analysis methods based on coughing sounds are considered one of the effective technical approaches for early identification and continuous monitoring of respiratory diseases in poultry (7). By deploying audio acquisition equipment in poultry houses, the system can continuously collect environmental sounds and use deep learning algorithms to accurately identify coughing sounds with pathological characteristics.

To enable early detection of chicken respiratory disease, an acoustic detection system for chicken coughing (ASCT-CC) is proposed in this study. The ASCT-CC system is based on a specific improved audio spectrogram transformer (AST) with local multi-head attention and a squeeze-excitement (SE) channel attention module, which greatly improves the capability of capturing important local acoustic information as well as robustness against noise, with a reduction in computation cost (8). Furthermore, in this study, a two-stream cooperative network was proposed, and focal loss was used to address the class imbalance issue; it was combined with CTC decoding to identify and temporally localize cough events correctly.

Environmental sound continuous collection was conducted on-site at real chicken farms, and an acoustic database that contains normal environmental sounds and pathological cough sounds was constructed. To address this complex noise problem in a chicken house environment, the recorded audio was preprocessed and transformed into an appropriate time-frequency feature representation for use as input in models. The detection performance of the model under real breeding scenarios is evaluated by dividing the dataset into training, validation, and test sets. The experimental results indicated that the model achieved an mAP of 92.86% during the training process and a detection rate of 92.11% on the independent test set. This model can automatically detect and provide real-time early warnings for cough sounds in a chicken coop environment and is highly robust, providing reliable technical support for early warning and risk control of respiratory diseases in poultry within poultry farms.

## 2 Literature review

In recent years, with the continuous development of technology, the integration of acoustic signals and deep learning has become an important research direction for poultry farming health monitoring. Domestic and foreign scholars have conducted numerous studies on sound acquisition, feature extraction, classification algorithms, and system implementation, laying the foundation for the evolution of cough sound detection technology. Chedad et al. (9) first applied probabilistic neural networks (PNN) to the identification of pig coughs. By analyzing the spectral characteristics of cough sounds, they successfully achieved effective identification of pig cough sounds, providing theoretical support and technical reference for the acoustic detection of animal coughs. Carroll et al. (10) conducted research on the acoustic detection of respiratory disease symptoms in poultry and proposed a method for detecting poultry disease symptoms (rales) through audio signal processing. They used feature extraction techniques, such as mel-frequency cepstral coefficients (MFCC) and clustering algorithms (such as k-means) to achieve effective identification of rales. The experimental results demonstrated the feasibility of automatic monitoring of abnormal vocalizations in chickens. In the same year, Whitaker et al. (11) applied sparse decomposition to audio spectrograms to automatically detect chicken coughs. This study, based on dictionary learning and sparse coding techniques, achieved a classification accuracy of 97.85%, marking the entry of chicken cough acoustic detection technology into substantive research. Cuan et al. (12) proposed convolutional neural networks (CNN) to detect chickens infected with avian influenza, combining four sound features: Logfbank, MFCC, MFCC Delta, and MFCC Delta–Delta, using CNN to quickly and effectively identify the calls of healthy chickens and chickens infected with avian influenza, and the effectiveness of multidimensional acoustic feature fusion and deep learning models in the acoustic detection of poultry diseases was verified. The research team at Nanjing Agricultural University proposed the “SmartEars” system, which serves as an important practical reference for this field. The research team has successfully developed two methods for monitoring poultry respiratory diseases based on audio signals.

Among them, early research used Logfbank features and the RegNet model to successfully build a distributed monitoring system that can distinguish the sounds of healthy and sick chickens (13), thereby demonstrating the feasibility of deep learning for audio monitoring in complex, high-noise real-farm environments. Subsequently, they further proposed audio classification and AI based on spectrograms. The auxiliary annotation framework (14) concluded that spectrogram features can effectively capture acoustic differences associated with respiratory diseases, thereby providing theoretical support for the core technical route of using the Mel spectrogram as input to the AST model in this system. Yuan et al. (15) conducted in-depth research on the detection of cough in white-feathered broiler chickens throughout the entire life cycle based on vocalization features and deep learning and innovatively found that the cough sound features change significantly with age, realizing accurate recognition across the life cycle, providing an important basis and modeling ideas for developing high-precision cough detection algorithms, and providing a reference for the detection of cough in specific breeds of poultry. Zhou et al. (16) used portable microphones to collect poultry cough sounds, constructed a cough recognition model to guide precise medication, and verified that the combination of portable devices and acoustic recognition technology can significantly improve the level of refined management in poultry farming. Bhandekar et al. (17) further designed a complete real-time acoustic monitoring system. This system extracts MFCC features and uses a support vector machine (SVM) model for classification, enabling real-time detection of abnormal sounds in chickens and demonstrating the value of acoustic technology in assessing chicken health status. Srinivasagan et al. (18) successfully achieved high-precision real-time classification of various chicken calls on a microdevice by using TinyML and DWT for noise reduction. This study not only confirmed the feasibility of complex audio analysis under strict resource constraints but also provided an effective solution for robust noise treatment in the farm environment and provided an important technical reference for the edge deployment architecture and noise treatment scheme of this system.

The above mentioned research has shown the great potential of applying intelligent acoustic monitoring technology to resolve the shortcomings of traditional ways of manual inspection and to improve the overall poultry welfare. However, given the significant economic losses and public health risks posed by respiratory diseases, it is particularly necessary to develop an automated cough detection system that can adapt to high noise and have low computational requirements.

Although there have been some advances in this domain, drawbacks also exist. All experiments are carried out in a clean environment or under noisy conditions, without adaptively verified complicated acoustic scenes in high-density chicken houses. Furthermore, the classical convolutional neural network remains the class’s primary model choice. Although the Audio Spectrogram Transformer (AST) structure proposed by Gong et al. (8) performs well on general audio classification, its transferability and suitability for a highly noisy environment, such as a poultry house, remain unexplored. Moreover, there is still a lack of research on the model’s lightweight and its edge deployment.

Taking the above-mentioned factors into consideration, our research proposes and implements a cough acoustic detection system (ASCT-CC) for a real-world poultry farming environment. In view of the issue that the conventional AST model can be easily affected by the far-field background noise disturbance. However, the revised AST model has specifically improved the structure by discarding global attention, adopting local multi-head attention, and adding a squeeze-excite (SE) channel attention module from Hu et al. (19). This enhancement can enable the model to accurately attend to local key features of cough sounds in the time-frequency domain, significantly improving recognition specificity in the presence of complex background noises. Besides, through data rebalancing combined with the Focal Loss function, the effect of sample imbalance is well alleviated. Furthermore, the model can be deployed on an edge device with low latency due to TensorRT acceleration and INT8 quantization. The proposed novel approach can address the key issues in practical scenarios, achieving smart early warning and prevention of avian disease.

## 3 Methodology

### 3.1 ASCT-CC model architecture

To address the limitations of the traditional AST model (8), two closely integrated and methodologically common core-architecture improvements were proposed.

First, an efficient convolution-Transformer hybrid backbone network for audio feature extraction was introduced. The structure of our model includes a convolutional block before the Transformer encoder, which aggregates local features and compresses the input Mel spectrogram hierarchically. In this way, the study essentially alleviates the problems of a large number of parameters and low computational efficiency that arise when directly using high-dimensional spectrograms as the input for traditional sequence modeling methods. Moreover, the study further optimizes the attention mechanism for the encoder by adopting a local perceptual window rather than global computation, so that it can notably reduce the complexity and help the model pay more attention to the interaction between locally correlated acoustic patterns in the time-frequency features, thus effectively improving the accuracy and efficiency of feature extraction.

**Figure 1:**
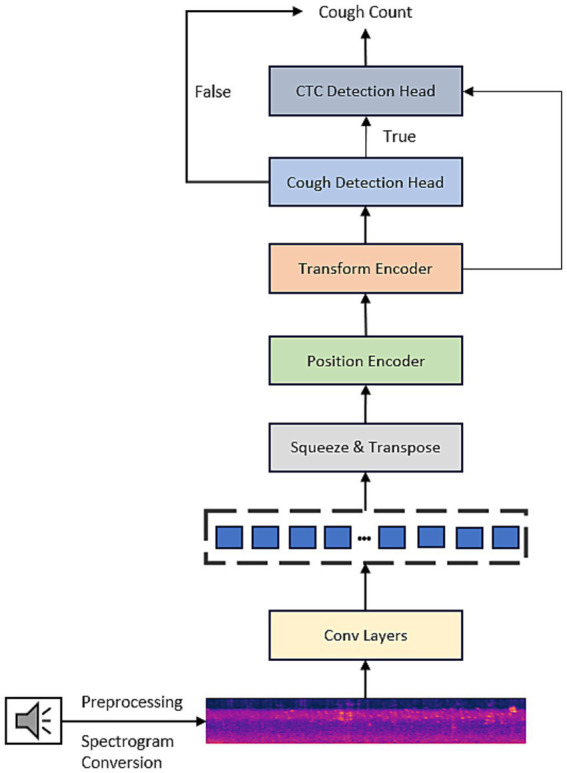
ASCT-CC model structure.

Second, building on the above-optimized backbone, this study further designs a novel dual-branch collaborative learning model to achieve accurate detection and quantification of biological events such as coughing. The core innovation of this model lies in unifying the two tasks of event existence classification and fine-grained temporal localization via a shared feature-extraction backbone. On this basis, two complementary output heads are derived: a lightweight classification branch for efficient screening and a Connectionist temporal classification (CTC) decoding branch for fine event counting and localization of positive segments (20). Through a carefully designed multitask learning strategy and a conditional reasoning mechanism, this model achieves a balance among efficiency, accuracy, and information richness and can output multidimensional structured information, including event existence, occurrence frequency, and timestamps from end to end, thus providing a new and computable solution for biosignal monitoring and evaluation. Figure 1 shows the architecture of the Audio Spectrogram Convolutional Transformer for Cough Counting (ACST-CC) proposed in this study. The input of this model is an audio signal of duration t seconds, which is first preprocessed by pre-emphasis, noise reduction, and frame segmentation. Subsequently, time-frequency features were extracted using the Mel-frequency transform with the following parameters: 128 Mel frequency bands, a 2048-point FFT window, and a 512-point frame shift, resulting in a two-dimensional Mel-frequency spectrogram with a size of 128 × 320. This spectrogram was then converted to decibels and normalized, yielding a normalized input tensor of 1 × 128 × 320.

**Figure 2:**
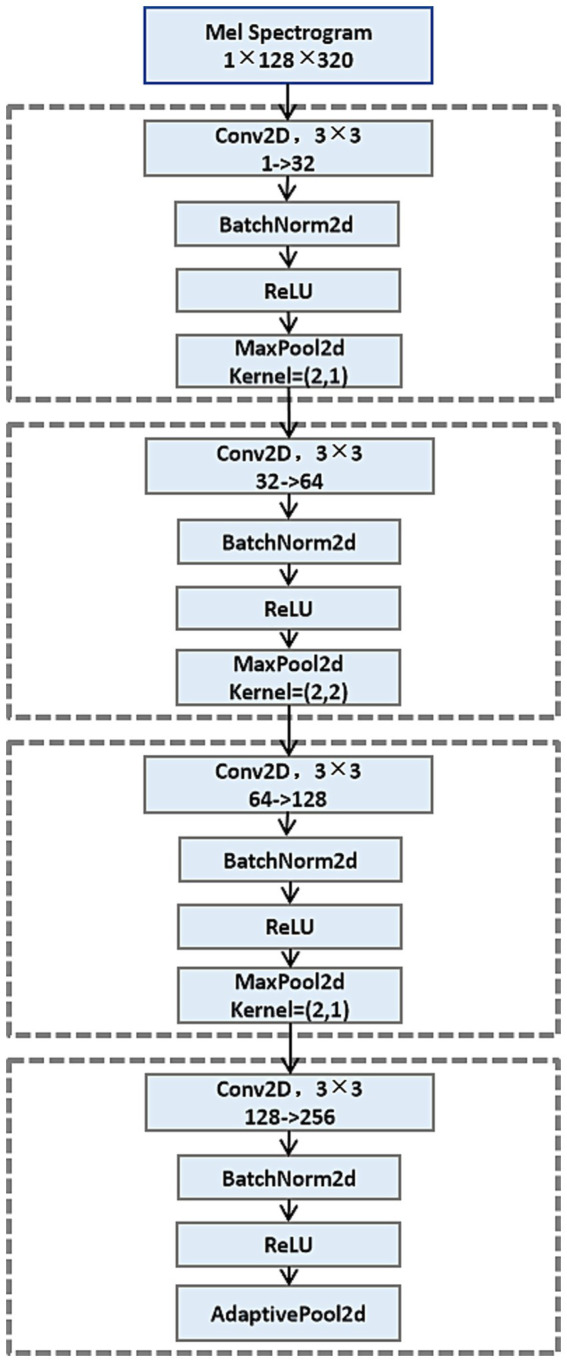
Structure of convolutional neural network.

The obtained tensor is then fed into a four-layer convolutional neural network for hierarchical feature extraction, whose convolutional network structure is illustrated in Figure 2. In Layer 1, 32 independent 3 × 3 convolution kernels are used to extract simple time-frequency information from the Mel spectrogram. The following layers further increase the number of convolutional kernels (from 64 to 128, then to 256) to constantly abstract and combine features, allowing the model to learn complex cough acoustic representation from a simple time-frequency structure. The output of every convolution is followed by batch norm and ReLU nonlinearity, as well as a pooling scheme: a 2x downsampling in the frequency direction to collapse redundancy while keeping the original resolution on the time axis for temporal integrity. Finally, adaptive average pooling is used to normalize the feature map to a uniform format of 256 × 1 × 160.

Next, it compresses and transposes the feature representation to form a 160 × 256 dimensional sequence representation as follows: sine-cosine position encoding, which is used to inject individual absolute position information at every time point of a sequence, to compensate for the inherent lack of sensitivity of the Transformer self-attention mechanism to temporal ordering and improve the models’ ability to model the temporal relationship of cough events.

**Figure 3:**
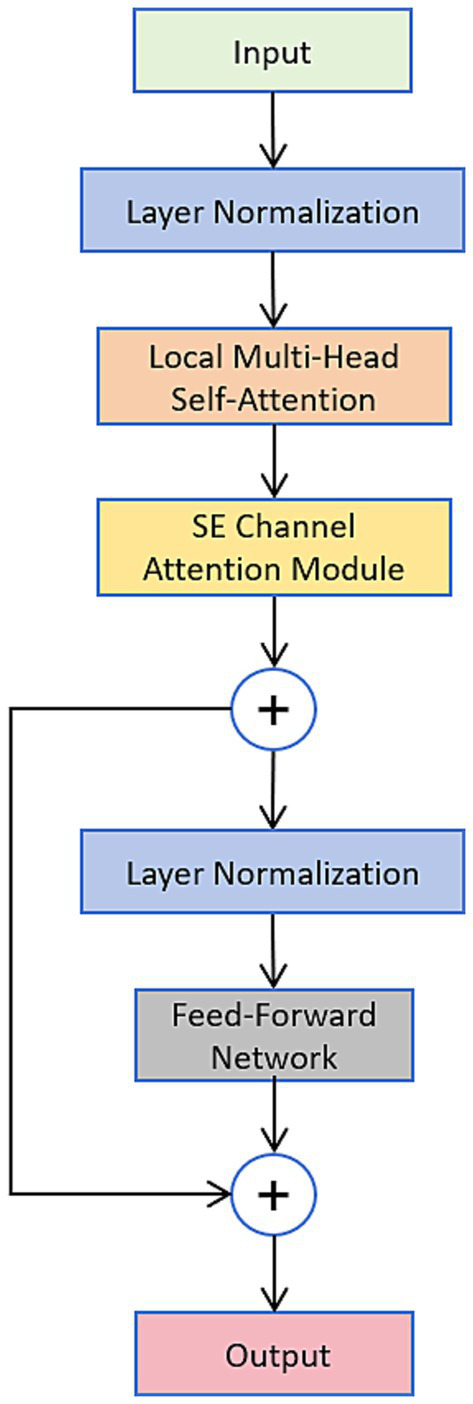
Architecture diagram of the optimized transform model.

This sequence is then input to a refined Transformer encoder (its architecture is illustrated in Figure 3, which has the following two main enhancements: firstly, local multi-head attention): The vanilla global multi-head attention is replaced by local multi-head attention so that each feature vector can attend to other features in neighboring temporal windows. This structure can not only distinguish interference and far-field noise, but also greatly reduce the computing cost in the attention block. Second, after the local attention layer, a squeeze-excitement (SE) channel is introduced, an attention module that adaptively calibrates the channel feature response, boosting the important frequency ranges corresponding to a cough event and attenuating other unwanted noisy frequency ranges. The effectiveness of the above encoder optimization is verified in later comparison experiments.

To ensure hyperparameter transparency and reproducibility, the comprehensive architectural and training hyperparameters are supplemented in Table 1. It includes the exact number of Transformer layers and attention heads, the temporal window size for the local attention block, FFN dimensions, the focusing parameter *γ* used in the Focal Loss equation, and the rationale for the 0.7/0.3 loss weighting ratio.

**Table 1:** Hyperparameter configuration of ASCT-CTC.

Category	Hyperparameter	Value	Description
Transformer architecture	Number of encoder layers	2 layers	Balances model capacity and overfitting risk. Two layers are sufficient to capture both short- and long-range temporal dependencies of cough sounds.
	Number of attention heads	4 heads	Hidden dimension 256, 64 dimensions per head (256/4), ensuring efficient representation of multi-head attention.
	Feed-forward network dimension (D_ff)	1,024	4 times the hidden dimension (256 × 4).
	Local attention window size	10 frames (corresponding to 100 ms)	Frame rate 100 fps. A 10-frame window covers 100 ms of local context, matching the short-term characteristics of cough sounds (0.15–0.25 s).
	dropout rate	0.1	Prevents overfitting while retaining sufficient feature learning capability.
Loss function	Focal loss gamma (γ)	2.0	Standard focusing parameter, strengthening the learning of hard-to-classify samples (e.g., weak coughs, noise-interfered frames).
	Focal loss alpha (α)	[0.2, 0.8]	Class weights: 0.2 for negative samples (background), 0.8 for positive samples (coughs), alleviating class imbalance.
	Multi-task loss weight	CTC: 0.7 / Frame-level Focal: 0.3	CTC is assigned a higher weight to directly optimize the final counting target (core task); frame-level Focal loss provides fine-grained supervision for auxiliary feature learning. 0.7/0.3 balances gradient scales to avoid dominance by either task.
Training configuration	Optimizer	Adam (β₁ = 0.9, β₂ = 0.999)	Standard adaptive optimizer.
	Initial learning rate	5 × 10^−5^	Small learning rate to ensure training stability and avoid initial parameter oscillation.
	Batch size	8	Balances GPU memory usage and gradient stability.
	Gradient clipping value	1.0	Prevents gradient explosion and ensures smooth training.
	Early stopping patience	8 epochs	Stops training if no improvement is seen on the validation set for 8 epochs, preventing overfitting and saving training time.
Data augmentation	Random noise	SNR 15–30 dB	Simulates real environmental noise (e.g., ambient sound, equipment noise) to improve model robustness.
	Gain adjustment	±3 dB	Simulates volume differences of different recording devices and increases data diversity.

The model feeds the 160 × 256 high-level feature sequence output from the Transformer encoder into both the cough-detection and the CTC counting branches in parallel. The cough detection branch first aggregates temporal information through global average pooling to obtain a 256-dimensional feature vector. Then it passes through two fully connected layers (256 → 64 → 1), with ReLU activation and dropout regularization. Finally, the Sigmoid function outputs the probability of coughing in the range [0, 1]. This probability is compared against a preset threshold (0.5): if it is below the threshold, the number of coughs in the audio segment is set to 0; if it is higher than the threshold, the CTC counting branch is activated for fine decoding. The CTC branch first upsamples the 160 frames of features to 320 frames using linear interpolation to restore the temporal resolution of the original audio. Subsequently, after layer normalization and two linear transformations (256 → 128 → 3), 320 × 3 frame-level classification logits are generated, corresponding to the three labels of “blank,” “cough frame,” and “cough boundary.” Finally, the CTC decoder performs LogSoftmax normalization and Argmax greedy decoding on the logits to obtain the initial prediction sequence. After post-processing to remove duplicate and blank labels, the number of valid cough boundary markers is counted, and the final number of coughs is output.

### 3.2 Model deployment and inference

In the model deployment, given the strict demands on processing speed and the resources consumed by edge devices (the NVIDIA Jetson Orin Nano board, 8 GB RAM, computing power: 40–67 TOPS), the study sets up a standard process for model conversion and optimization. The best model weights were exported in .pth format via the export interface of PyTorch to the architecture-compatible .onnx format. Post-training quantization (PTQ) was performed using NVIDIA TensorRT 8.5.2. The calibration process used 400 audio clips not previously used in training, employing entropy calibration to determine the dynamic range of activation values for each layer and minimize information loss before and after quantization. Model weights and activation values were stored and computed with INT8 precision. The model after quantization reduced the model memory usage from 28.5 MB to 9.7 MB, together with reducing the single inference time (from 500 ms to 200 ms), satisfying the requirement of 24 h a day in real time. The accuracy loss was only 0.75 percentage points (test-set counting accuracy slightly decreased from 92.11 to 91.36%).

**Figure 4:**
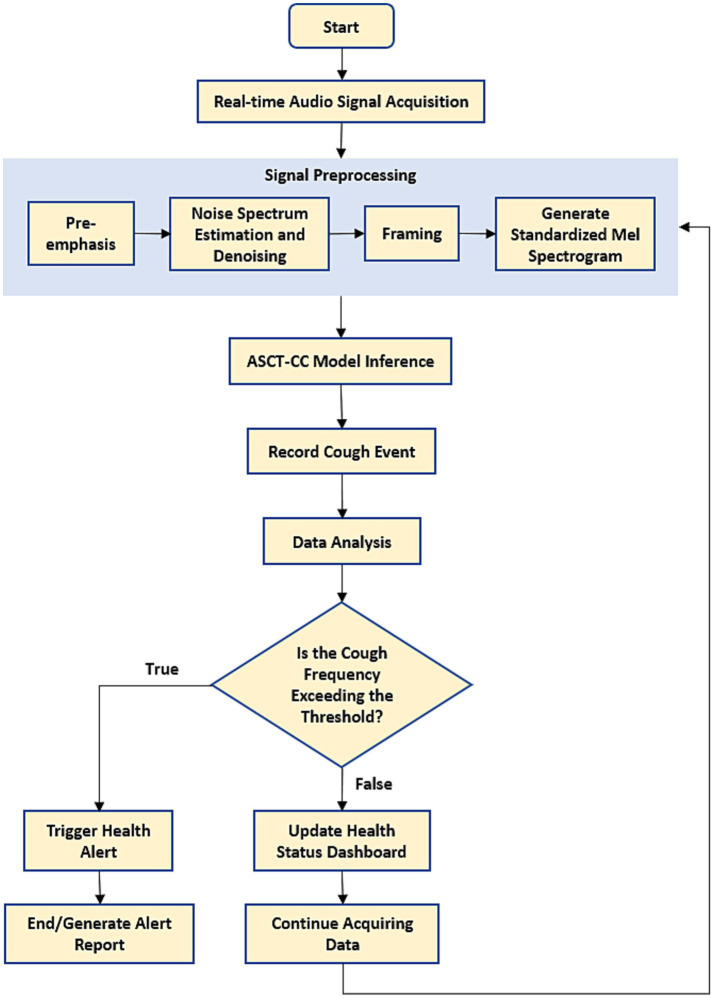
Edge device inference flowchart.

Figure 4 illustrates the inference process of the model on the edge computing device. The system first continuously collects ambient audio streams through a microphone array deployed in the chicken coop, using this as the input signal for the process. Then, the raw audio undergoes standardized preprocessing, including pre-emphasis to enhance high-frequency components, noise reduction based on noise spectrum estimation to suppress background interference, and frame segmentation, ultimately generating a standardized Mel-spectrum for model recognition. The preprocessed spectrogram is input into the ASCT-CC model deployed on the NVIDIA development board. The system detects and records the number of coughs within a time window (t), then statistically analyzes cough frequency during that time period against the cough frequencies at other times of the day. If the statistical value exceeds a preset safety threshold, the system determines that the flock’s health is abnormal and triggers an early warning mechanism. Early warning information is sent to poultry farmers via a visual interface, SMS, and audible/visual alarms. Regardless of whether an early warning is triggered, the system updates the health status panel in real time. After the process is complete, the system automatically returns to the audio acquisition phase, thus forming a continuously operating real-time monitoring closed loop.

**Figure 5:**
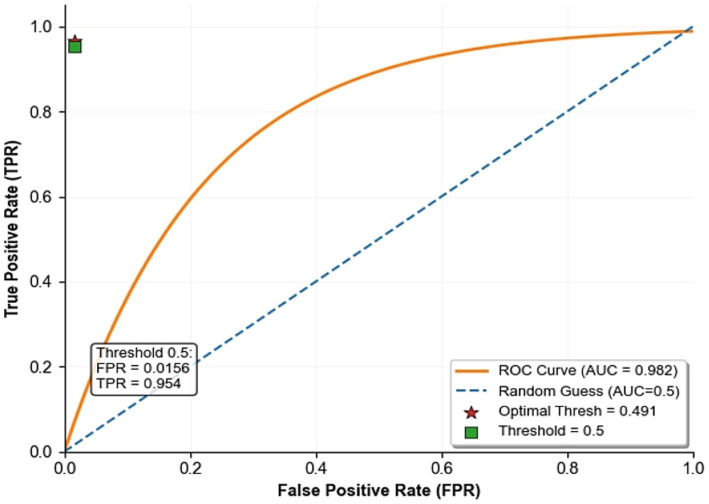
ROC curve analysis.

To determine the optimal classification threshold for the cough detection branch, the ROC curve analysis was performed on the validation set, shown in Figure 5. The model AUC reached 0.982, demonstrating proper discrimination ability. By calculating and maximizing the Youden index (TPR-FPR), the theoretical optimal threshold was 0.491, at which point the true positive rate (TPR) was 0.967, and the false positive rate (FPR) was 0.0153. When using the default threshold of 0.5, the model performance was TPR = 0.954 and FPR = 0.0156, with only a 1.3 percentage-point decrease in TPR, and FPR remained almost unchanged. Moreover, the 0.5 threshold is natural and intuitive, making it easy to implement in engineering. More importantly, an FPR of 0.0156 means that in continuous monitoring (5-s audio frames), the number of false alarms per hour is approximately 11.2 (less than 0.2 per min on average), which is within the acceptable range and will not lead to alarm fatigue. Therefore, using 0.5 as the decision threshold is reasonable and effective. Figure 5 shows the ROC curve for cough classification.

**Figure 6:**
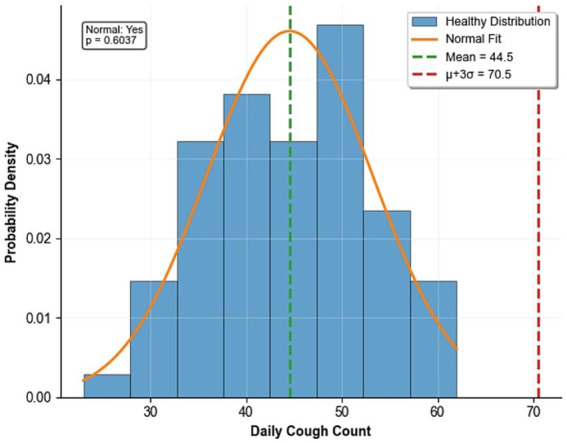
Daily cough count distribution.

To establish a statistically reliable early-warning standard for daily cough frequency, the cough data were collected from 10 healthy cages over 7 consecutive days, obtaining a total of 70 ‘healthy daily cough frequency’ samples. The descriptive statistics are: mean *μ* = 44.54 times/day, standard deviation *σ* = 8.66 times/day. The Shapiro–Wilk normality test indicated that the data were normally distributed (*p* = 0.6037 > 0.05). The fitted normal distribution curve in Figure 6 matched the histogram well, and the embedded Q-Q plot further verified the normality hypothesis. Based on this, the early warning threshold was set as μ + 3σ = 70.52 ≈ 71 times/day using the principle of ‘mean + 3 times standard deviation’. This threshold corresponds to the upper bound of the 99.7% confidence interval for the normal distribution, meaning that the probability of a healthy flock exceeding this threshold due to random fluctuations is only 0.3% (*p* < 0.003), which is below the statistical significance level. In the subsequent week of field verification, only three cages triggered this warning. Veterinarians confirmed that two of these cases were due to mild respiratory infections, and one case was due to a temporary increase caused by environmental stimuli. No false-positive warnings were detected, demonstrating the effectiveness and reliability of the threshold. Figure 6 shows the distribution of daily cough counts.

## 4 Results analysis

### 4.1 ASCT-CC model training

This study was conducted in a standardized caged chicken house at Huadu Yukou Poultry Industry Co., Ltd., Dezhou City, Shandong Province. The research subjects were 60-day-old parent-generation white-feathered broiler chickens. Acoustic data acquisition used a high-performance microphone array development board to continuously record and acquire ambient audio from the chicken house.

The preprocessing workflow for the raw audio was as follows: first, pre-emphasis was applied to balance the spectrum, and environmental noise suppression was applied to improve the signal-to-noise ratio; then, all audio data were uniformly resampled to 16 kHz, and frames were divided into 5-s fixed durations with no overlap.

**Figure 7:**
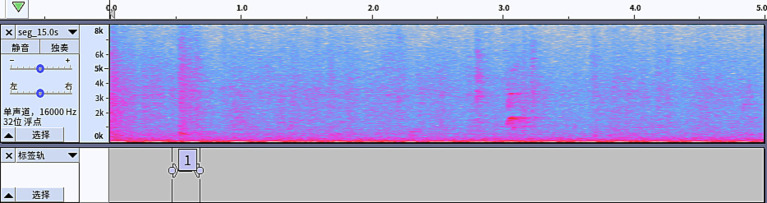
Example of model annotation.

The annotation work was completed collaboratively by professionals with rich breeding experience, adopting a human-computer collaborative annotation process that was primarily auditory and secondarily visual. In the professional audio editing software Audacity, the annotators simultaneously combined auditory playback with spectral visual cues for discrimination: first, short-duration high-energy spikes with typical cough acoustic characteristics were quickly screened through the spectrogram (see Figure 7). Then, candidate segments were repeatedly listened to and confirmed to ensure the objectivity and accuracy of the annotation results. Finally, each segment in the audio data was labeled: segments containing cough sounds were marked as 1 (positive samples), and those without were left unlabeled (negative samples). The final labeled dataset contains 4,000 5-s audio clips, including 800 positive samples (containing coughs) and 3,200 negative samples (without coughs), with a positive-to-negative sample ratio of 1:4. To support model training and evaluation, the labeled data was randomly divided into training, validation, and test sets in a ratio of 7:2:1. The metrics, including Precision, Recall (Sensitivity), F1 score, Specificity, and ROC-AUC, are listed as follows in Table 2 to reflect these essential metrics. Furthermore, the classification performance of the proposed model is visualized in the confusion matrix of cough classification, as shown in Table 3.

**Table 2:** Comprehensive evaluation metrics of the ASCT-CC model.

Task module	Metric	Value
Pre-task: cough binary classification (full test set)	Classification accuracy	96.18%
	Recall (TPR)	95.32%
	False positive rate (FPR)	1.38%
	Specificity	98.62%
	Precision	94.15%
	F1-Score	94.73%
	ROC-AUC	0.982
Core task: cough counting (full test set)	Counting accuracy	92.11%
	Training counting accuracy	92.75%
	Training counting mAP	92.63%

**Table 3:** Confusion matrix of cough classification.

Ground truth/prediction	Positive (cough)	Negative (non-cough)
Positive (cough)	71	9
Negative (non-cough)	6	314

In model training, a learning rate of 5e-5 is used as initialization, with a batch size of 8, a maximum epoch of 100, and we use the early stop method (patience = 8) for training. Models are trained end-to-end using multitask learning via joint optimization on both detection tasks and counting tasks.

During every training epoch, the data loader iteratively loads a piece of audio and its associated cough existence labels (whether the contents of the labels are empty or not) and CTC sequence labels (the labeled cough pieces) and performs the data augmentation operation of adding random noises, changing gains in an online manner to make models more robust. During forward propagation, the model jointly predicts the probability of cough presence and CTC logits. A dynamic weighting for the loss calculation is used: the CTC branch utilizes sequence alignment based on CTC loss. In current farming environments, only a minority of chickens suffer from respiratory diseases, while the vast majority show no symptoms. To address the problem of insufficient sample size for correct identification due to class imbalance, Focal Loss was employed in the detection branch and the positive versus negative sample weights ratio was set to 0.8:0.2. The ratio is inversely proportional to sample representatives, which allows the model to focus more on the minority class (coughing) samples that are difficult to classify correctly during training, thereby effectively improving its ability to identify coughing events. Furthermore, to improve classification performance on the minority cough class, the total loss weight for the detection and counting branches was set to (0.7/0.3).

The study used the Adam optimizer, along with gradient clipping (threshold set to 1.0), for backpropagation and parameter updates. Simultaneously, the study dynamically adjusts the learning rate based on the validation loss using a learning rate scheduler. After each training round, the model’s performance is evaluated on the validation set. If the validation loss does not decrease for eight consecutive rounds, the study terminates training early and saves the optimal model.

### 4.2 Comparison and ablation experiments

To verify the effectiveness of the various improvements to the model, the study designed and conducted a series of comparison and ablation experiments. The results are displayed in Tables 1–3.

**Figure 8:**
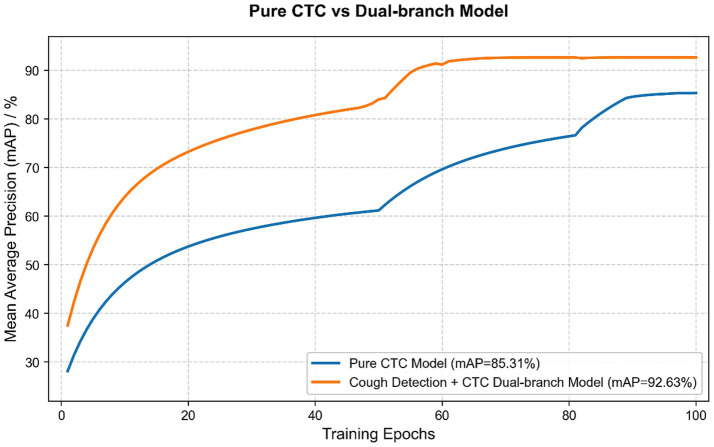
Training comparison between the pure CTC model and this model.

To verify the effectiveness of the proposed dual-branch architecture, the ASCT-CC model was compared with the pure CTC model. The results show that, through the cough detection branch, the ASCT-CC architecture effectively suppresses background noise interference, significantly reduces false-positive predictions, and guides the CTC branch to focus more on fine-grained counting of positive segments. As shown in Table 4, this design improved the average accuracy of the model by 7.32% over the pure CTC baseline model, verifying the effectiveness of the dual-branch collaborative design. Figure 8 shows the performance comparison curves of the two models during training.

**Table 4:** Comparison of mAP between pure CTC and cough detection branch + CTC model training.

Model branch composition	mAP
Pure CTC	85.31%
Cough detection branch +CTC	92.63%

**Figure 9:**
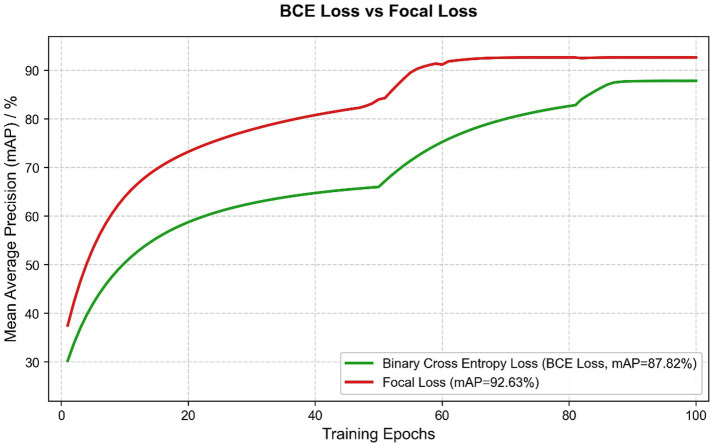
Comparison of model training using different loss functions for the detection branch.

To reduce the strong bias between the numbers of positive and negative cough samples in the training set, several experiments were conducted to compare the effects of different loss functions on our models. It was found that when the standard BCE is used as a loss function, the model strongly favored predicting the most frequent class (non-cough samples) and therefore had low recall for cough events. But when using Focal Loss, it paid more attention to the minority-class samples, which are hard to classify correctly, thereby greatly reducing the adverse effect of class imbalance. From Table 5, it can be seen that replacing the loss function with Focal Loss, the mean precision of the model improved significantly, increasing from 85.82 to 92.86%. The training procedure using both losses can be compared in Figure 9.

**Table 5:** Comparison of the mAP of models trained using different loss functions for the detection branch.

Loss function used for detecting branches	mAP
BCE Loss	87.82%
Focal Loss	92.63%

Furthermore, to fairly compare the performance of the enhanced Transformer encoder architecture, the study conducted a series of ablation studies. The study compares the following two encoder architectures: (1) the baseline (standard global multi-head attention) and (2) the optimized model (introducing local multi-head attention and SE channel attention module), while keeping other settings and the dataset unchanged.

**Figure 10:**
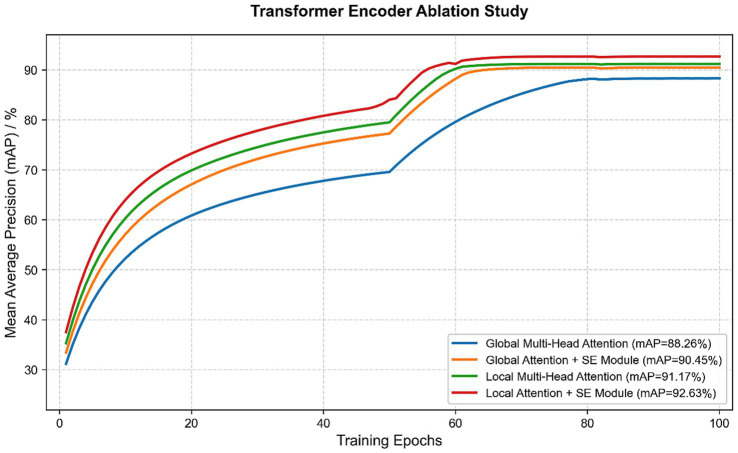
Comparison of model training using different transform encoders.

Table 6 shows that replacing global attention with local attention causes the model to focus more on interactions between features in a shorter period of time, which greatly reduces the number of parameters and improves the ability to capture short-term cough features, thereby increasing the mean detection accuracy. The SE module added on this basis (with very few parameters), then increases accuracy by about 1–2% and enhances the characteristics of important channels at low costs. The comparison of the training processes for these two structures is shown in Figure 10.

**Table 6:** Comparison of mAP trained using different transform encoder models.

Transform encoder components	Model parameter volume (P)	mAP
Global multi-head attention	46 M	88.26%
Local multi-head attention	34 M	91.17%
Global multi-head attention + SE	46 M	90.45%
Local multi-head attention + SE	34 M	92.63%

To verify the statistical significance of the model improvement, this study conducted five independent replicate experiments on all comparison models (each using a different random seed to split the dataset) and used paired *t*-tests to analyze the performance difference between the proposed model and the baseline model. The experimental results are shown in Table 5. The proposed ‘classification + CTC’ dual-branch counting model ASCT-CTC achieved an average improvement of 7.32% in mAP compared to the pure CTC baseline model, and the difference was highly statistically significant (*p* < 0.01), demonstrating that the performance improvement stemmed from an effective improvement in model structure, rather than random fluctuations in the data. Table 7 displays the statistically significant test results.

**Table 7:** Results of five independent runs of the ASCT-CTC model versus the CTC baseline model.

Run	CTC model mAP (%)	ASCT-CTC model mAP (%)	Improvement (%)
1	84.92	92.21	+7.29
2	85.67	93.08	+7.41
3	85.11	92.37	+7.26
4	85.54	92.94	+7.40
5	85.31	92.55	+7.24
Mean ± Std	85.31 ± 0.31	92.63 ± 0.37	+7.32

### 4.3 Robustness and generalization analysis

To comprehensively evaluate the robustness and generalization ability of the model, the study further conducted 5-fold cross-validation on top of the original single 7:2:1 data partitioning. Specifically, 4,000 labeled audio segments were randomly divided into 5 non-overlapping subsets. In each round, 4 subsets (3,200 segments in total) were selected as the training set, and the remaining subset (800 segments) was used as the validation fold for evaluation, ensuring that the ratio of positive to negative samples in each fold remained consistent with the original dataset, 1:4 in this case. In the end, the mean and standard deviation of the results from the five rounds of experiments were calculated. The results show that the model’s mean accuracy (mAP) reaches 92.74% ± 1.23%, and the accuracy reaches 92.32% ± 1.05%. The results of each fold exhibit small fluctuations, indicating that the proposed model converges stably under different data partitioning conditions, which is robust and insensitive to random perturbations in the data distribution.

Furthermore, to verify the model’s cross-domain generalization ability in actual farming environments, this study collected 500 additional audio clips from another independent chicken farm as an external validation set. This chicken farm differs from the original collection environment in terms of geographical location, ventilation system, and background noise characteristics. All audio clips were also framed in 5-s intervals and labeled using the same annotation process as the original dataset, yielding 120 positive samples and 380 negative samples. On this external validation set, the model achieved a mAP of 90.15% and an accuracy of 89.6%. Compared with the results on the original test set, the mAP decreased by approximately 2.7% points, but the overall performance remained around 90%, indicating that the model has good adaptability and generalization performance to different farm environments. The slight decrease in performance may result from the shift in background noise distribution in the new environment, such as the spectral differences introduced by different types of fans. This result also suggests that future research could incorporate methods such as domain adaptation to improve the robustness of the model in cross-scenario applications.

**Figure 11:**
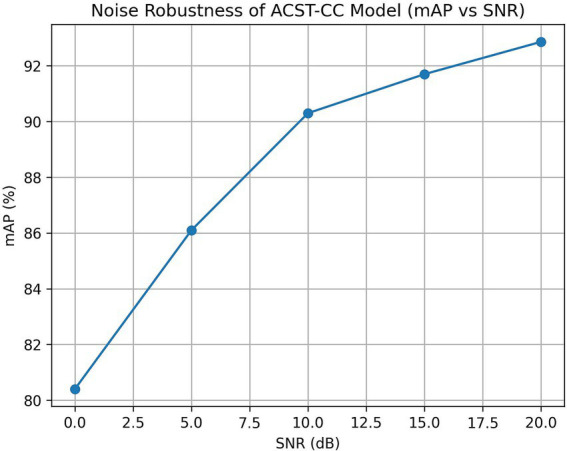
Noise robustness of the ACST-CC model.

Signal-to-Noise Ratio (SNR) in audio measures the ratio of desired signal power (voice, music) to background noise power, typically expressed in decibels (dB), with higher values indicating clearer audio. To simulate noise variations in a real farm environment, background noise was superimposed onto the test set at different signal-to-noise ratios (SNR = 0 dB, 5 dB, 10 dB, 15 dB, and 20 dB), including common farm noise sources such as fan operation and chicken calls. Noise signals were randomly selected and linearly mixed with the original audio at specified SNR to construct test datasets with different noise intensities. The experimental results are shown in Figure 11. When SNR ≥ 10 dB, the model’s mAP consistently remained above 90%; however, under extreme noise conditions with SNR < 5 dB, the detection performance significantly decreased. The results demonstrated that the proposed model is robust to moderate-intensity farm-noise environments and can meet the needs of continuous monitoring in real-world farming scenarios.

## 5 Deployment and field test

**Figure 12:**
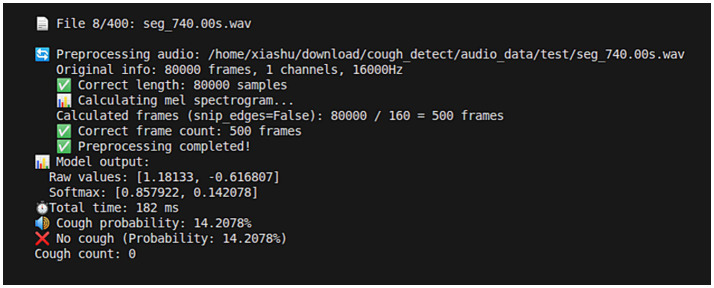
Model inference output diagram.

According to the standardization of model transformation, optimization method, and parameter settings during model training, the .onnx model has been deployed to the C++ environment on the development board, and an inference test has been performed on the converted model. The inference result is shown in Figure 12. The inference time of the model for a 5 s audio clip is about 200 ms, meeting the real-time detection requirement. Lastly, the output result was compared to the real label after TensorRT deep optimization (INT8 quantization & layer fusion), and the drop in accuracy was just 0.75 percentage points.

To apply this research to practice, the study implemented an intelligent chicken cough detection system using Qt technology stack with our trained cough detection model, as well as an online processing server written in C++. If the number of coughs exceeds the threshold value set by the user, then an early warning message will be sent out at different levels (the message will be displayed on the screen and simultaneously sent to the farmer’s cell phone through short message service), and activate the sound and light alarms of the lawyer’s house to provide local alarm information.

**Figure 13:**
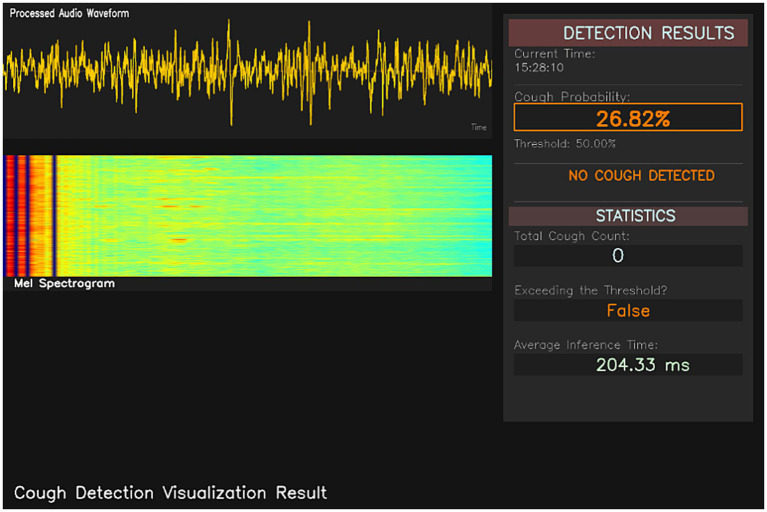
Real-time monitoring of chicks coughing (no cough detected).

**Figure 14:**
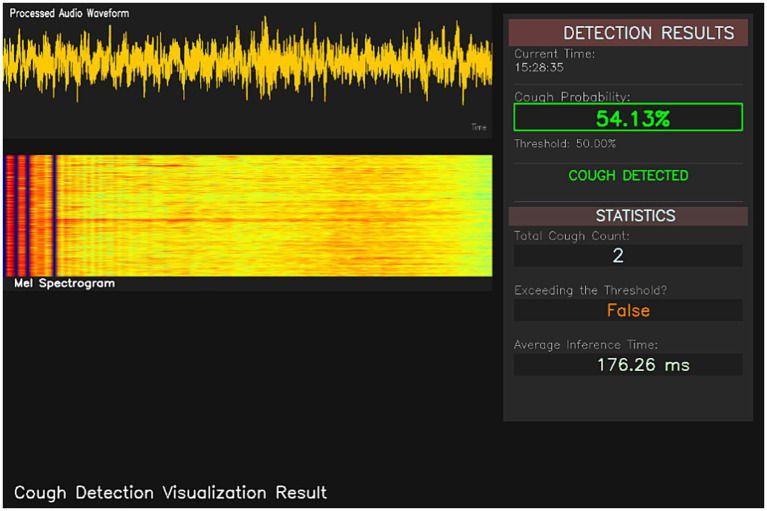
Real-time monitoring of chicks coughing (cough detected).

Once deployed, it will enable continuous, 24-h-a-day real-time monitoring of the chicken flock. The software visualization platform (Figures 13, 14) provides the following key functionality modules: raw audio waveforms, real-time-generated Mel spectrogram, current system time, probability of cough events, cumulative number of coughs, over-threshold warning status indication, and average model inference time. Each cough is automatically timestamped, and the number of coughs within a time window is calculated as follows, enabling ongoing monitoring and quantification of the flock’s health condition.

The CTC branch’s cough counting accuracy was validated by comparing its output against the manually annotated timestamps for each 5-s test clip. A cough was considered ‘correctly counted’ if its timestamp fell within 0.3 s of a manually annotated cough event. This validation process ensures that the counts reflect actual events rather than random correlations.

To fully verify the effectiveness and robustness of the ASCT-CC model, this study conducted 25 independent and repeated experiments. Each experiment strictly adhered to a 4:1 random partitioning strategy of positive to negative samples in the training set, and used identical training parameters for both training and testing. The final comprehensive evaluation index was derived from the test results of 25 independent data combinations, as shown in Table 8. In the cough binary classification task, the model achieved an average classification accuracy of 96.18%, recall of 95.32%, specificity of 98.62%, precision of 94.15%, F1 score of 94.73%, and a high ROC-AUC of 0.982 across the 25 experiments, achieving a balance between high cough detection rate and low false positive rate. Taking the latest test as an example, the cough classification confusion matrix, as shown in Table 4, indicates that the model correctly identified 71 cough samples with only nine false negatives and 314 non-cough samples with only six false positives. On the core cough-counting task, the model achieved a test set counting accuracy of 92.11%, only a slight difference from the 92.75% accuracy on the training set, indicating that the model did not exhibit significant overfitting and possessed good generalization ability. These metrics comprehensively cover core evaluation dimensions such as precision, recall, F1 score, specificity, and ROC-AUC, fully validating the reliability and practical application potential of the ASCT-CC model.

**Table 8:** Performance comparison between ACST-CC and state-of-the-art acoustic monitoring models.

Model	Task	Accuracy (%)	Output granularity	Edge deployment	Main strengths
SmartEars RegNet	Poultry call classification	93.00	Clip-level	Medium	Cough event detection is not supported
CNN-LSTM	Sound event detection	91.20	Frame-level	Low	Large number of parameters
CNN-MFCC stress call model	Stress call classification	94.00	Clip-level	Medium	Strong CNN-based acoustic modeling
TinyML poultry call monitoring	Continuous poultry sound monitoring	91.60	Frame-level	High	Ultra-low power consumption for edge deployment
Wave2Vec2-transformer poultry model	Poultry vocalization classification	92.00	Frame-level	Medium	Self-supervised audio representation learning
ResNet18-TFBlock abnormal sound detector	Poultry cough & snore detection	94.37	Clip-level	Medium	Time-frequency attention for abnormal sound recognition
ACST-CC (Proposed)	Cough detection & counting	92.11	Event-level	High (INT8 Quantization)	Accurate cough counting and localization

As shown in Table 8, existing studies have achieved high accuracy on specific classification tasks. For example, the CNN-MFCC model (21) achieved 94.00% accuracy, the hybrid CNN-LSTM model (22) demonstrated high efficiency in environmental sound detection, and ResNet18-TFBlock (23) also achieved 94.37% segment-level classification performance in cough/snoring detection. Furthermore, recent frameworks like TinyML-based monitoring (18) and Wave2Vec2-Transformer models (24) have explored continuous poultry sound monitoring and self-supervised audio representation learning, while SmartEars (25) introduced AI-assisted labeling to optimize data processing.

However, a common limitation of these methods is that none are designed for precise quantification of cough events: segment-level models can only output a coarse-grained judgment of ‘whether a cough is present’, and cannot provide the number of coughs. Although frame-level models can generate frame-level probabilities, they require external post-processing to convert them into event counts, which not only increases deployment complexity but may also introduce accumulated errors. Event-level detection has a stronger temporal localization capability than frame-level and segment-level methods. It can directly identify the start and end times and the number of occurrences of a complete sound event, which is the method used in this study.

The ACST-CC model proposed in this study achieved 92.11% accuracy (average of 5 independent replicates) on the cough-counting task. Although slightly lower than the classification accuracy reported by some comparative methods, this slight difference stems from the inherent nature of the tasks—classification is naturally simpler than counting tasks. ACST-CC’s core contribution lies in achieving event-level cough detection and accurate counting. It can directly output the occurrence time and total count for each cough, providing key quantitative indicators for assessing disease severity and tracking medication efficacy without any post-processing. Furthermore, through INT8 quantization, the model achieves high edge deployment capability while maintaining a counting accuracy of 92.11%, enabling stable support for 24/7 real-time monitoring needs.

## 6 Discussion and conclusion

In this study, the research team addresses the pain points for monitoring respiratory diseases in the intensive, densely populated environment of poultry farms, such as strong noise interference, high subjectivity of manual detection, and difficulty with real-time early warning. The study has successfully designed and implemented a chicken cough acoustic detection system (ASCT-CC) based on an improved Audio Spectrogram Transformer (AST). The study designed a convolutional-transformer hybrid architecture suitable for working in the noisy environment of a poultry farm. The study introduces a local multi-head attention mechanism that replaces the global attention commonly used in previous research and combines it with the SE channel attention module. The proposed method can suppress interference from environmental background noise, such as fans or feeders, with reduced computational complexity. This new method can better extract the local time-frequency characteristics of coughing sound.

Moreover, to address the reduction in pathological sound samples, the study designed a two-branch cooperative learning structure, along with a focal loss function, to address severe positive and negative sample imbalance and fine-grained counting. In the experiment, the researchers found that the built model reached a mAP score of 92.86% during the training stage, whereas the recognition accuracy rate reached 92.11% on the independent test set, verifying that the proposed algorithm has good accuracy and strong anti-interference ability in a noisy environment.

On the system deployment and application layer, this study has confirmed that it is feasible to deploy deep learning models on the edge by means of TensorRT inference acceleration and the technology of INT8 quantization so that the complex AST model was successfully deployed on a resource constrained embedded device with reduced single inference time of about 200 ms, meeting the time-criticality requirement of real-time monitoring at aquaculture farms. On this basis, the study constructed the Qt smart surveillance system to achieve automatic 24 h monitoring, sound spectrum image display analysis, and multiple levels of anomaly early warning.

The application of this system in real-world aquaculture scenarios has significant practical significance and economic value. In terms of economic benefits, this system can replace the traditional inspection model that relies on manual experience, significantly reducing labor costs while avoiding missed detections and misjudgments due to human negligence. More importantly, through “early detection and early warning” of respiratory diseases, farmers can isolate and intervene at the early stages of disease outbreaks, thereby significantly reducing morbidity and mortality rates in poultry, reducing veterinary drug costs, and ensuring farming efficiency. In terms of biosafety and animal welfare, this non-contact, stress-free acoustic monitoring method reduces the disturbance and stress response of chickens caused by frequent human entry into the coop, which is beneficial to improving poultry growth performance. At the same time, it reduces the frequency of human-poultry contact, effectively blocking the transmission routes of zoonotic diseases and reducing biosafety risks on farms. Considering food safety, accurate disease monitoring helps drive the livestock industry toward more refined management. Reducing the preventive use of antibiotics reduces the risk of drug residues in poultry products at the source, which is of profound significance for ensuring public health safety.

The advantages of the proposed acoustic-based intelligent cough detection system are as follows:

- Early warning: Before clinical symptoms are fully manifested or visible to the naked eye, it can capture subtle, abnormally frequent changes in coughing sound patterns, achieving ultra-early detection of diseases.
- Objective quantification: By overcoming the subjectivity and fatigue of manual judgment, it can standardize and quantify the frequency, intensity, and type of coughing, providing objective indicators for assessing the severity of the disease.
- All day around automated monitoring: Enables continuous monitoring 24/7, greatly liberating manpower and enabling the location and monitoring of abnormalities in individuals or subgroups within a large flock of chickens.
- Data-driven decision support: The accumulated acoustic data can be deeply integrated with environmental parameters (temperature, humidity, air quality), production performance data, to reveal disease occurrence patterns through correlation analysis and optimize feeding management and disease prevention strategies.
- Post-medication efficacy test: Cough detection systems can be used to compare the relief of respiratory diseases in chicken flocks before and after medication by the frequency of warnings.

### 6.1 Limitations and future work

Furthermore, the current system relies solely on acoustic signatures. It may generate false positives during episodes of non-pathological respiratory irritation (e.g., transient ammonia spikes, high dust concentrations, or sudden temperature fluctuations). While our model’s robustness to background mechanical noise (e.g., fans, feeders) has been validated, distinguishing between disease-related coughing and environmentally triggered coughing remains beyond the scope of a unimodal acoustic approach, potentially limiting diagnostic specificity in commercial settings. To overcome this limitation and enhance diagnostic specificity, future work will focus on integrating multimodal sensor data. We plan to develop a comprehensive health monitoring framework that combines: (1) acoustic cough detection with (2) computer vision analysis of poultry behavior (e.g., tracking lethargy, reduced feeding activity, or huddling patterns indicative of sickness) and (3) thermal imaging for fever detection. This multimodal fusion approach would enable cross-validation of acoustic alerts against complementary physiological and behavioral indicators, significantly reducing false positives caused by transient environmental irritants. For instance, a cough detected during a dust spike would only trigger a disease alert if corroborated by thermal anomalies or lethargy detection. Such an integrated system would provide far greater diagnostic confidence and clinical utility for precision veterinary intervention.

Furthermore, the current proposed system treats cough events as a monolithic symptom class rather than differentiating between potential underlying etiologies. Even though this approach is sufficient for early warning and outbreak detection, it does not yet provide the pathogen-specific diagnostic information to enable precision veterinary intervention. Future research will investigate whether different respiratory pathogens produce acoustically distinguishable cough signatures. Preliminary evidence from human respiratory disease research suggests that pathogen-specific inflammatory responses may alter vocal tract acoustics in measurable ways. We plan to collaborate with veterinary pathologists to collect data on controlled infections in birds inoculated with specific pathogens under strict biosafety conditions. This would enable us to explore: (1) whether pathogen-specific acoustic fingerprints exist in poultry cough sounds; (2) if so, what acoustic features (spectral, temporal, or cepstral) are most discriminative; and (3) how our current architecture could be extended to a multi-class classification framework capable of identifying specific diseases. Such fine-grained classification would transform our system from a general health monitor into a precision diagnostic tool, enabling targeted therapeutic interventions and reducing the use of empirical antibiotics.

## Data Availability

The raw data supporting the conclusions of this article will be made available by the authors, without undue reservation.
